# Removal of CD5 on T cells alters their differentiation and cytokine production in an *in vitro* model investigating effects of *P. gingivalis* LPS on oral epithelial and immune cells

**DOI:** 10.3389/fimmu.2026.1761775

**Published:** 2026-06-10

**Authors:** Carlos Moreno, Alina Svitlana Rodriguez Bezruchko, Dallin Cardon, Claudia M. Tellez Freitas, K. Scott Weber

**Affiliations:** 1 Brigham Young University, Department of Microbiology and Molecular Biology, Provo, UT, United States; 2 Roseman University of Health Sciences, College of Dental Medicine, South Jordan, UT, United States

**Keywords:** CD5, chronic inflammation, gingival epithelia, LPS, mucosal epithelia, periodontitis, Porphyromonas gingivalis, T cells

## Abstract

**Introduction:**

Periodontal disease is a highly prevalent oral inflammatory disease that affects nearly half of adults 30 years or older in the United States. It is characterized byexcessive inflammation within the periodontal pockets typically in response to bacterial challenge and is characterized by inflamed gums, destruction of periodontal ligaments, alveolar bone loss, and tooth loss if left untreated. T cells are adaptive immune cells which play important roles in driving inflammation and alveolar bone loss during severe periodontitis. Additionally, several studies have reported associations between periodontal pathogens and chronic inflammation within the oral cavity to several systemic diseases, including inflammatory bowel disease, diabetes mellitus, cardiovascular diseases, cognitive decline and Alzheimer’s disease, chronic obstructive pulmonary disease, and certain cancers. CD5 is a glycoprotein receptor found on the surface of T cells that serves as a coinhibitory receptor that attenuates TCR signaling, and its immunoregulatory role has yet to be investigated in the context of periodontitis.

**Methods:**

Here, we characterize the functional differences between CD5 knockout T cells and wildtype T cells, including T cell activation, differentiation, and cytokine production, in an *in vitro* model used to investigate the effects of *P. gingivalis* LPS on oral epithelial and immune cells.

**Results and Discussion:**

In this study we report that removal of CD5 increases T cell activation and effector/memory formation and increased CD4+ T cell Csf1 mRNA transcription while decreasing Rankl transcription. Together, these findings provide insights into the role of CD5 in modulating inflammation during periodontal disease.

## 1 Introduction

Severe periodontal disease (PD) is a chronic inflammatory oral disease that is characterized by the destruction of periodontal ligaments, alveolar bone loss, and inflamed gums (1–3). Despite PD being preventable by proper oral hygiene, it has a high prevalence and economic burden worldwide (4, 5). PD affects nearly half of adults 30 years or older in the United States, and the direct economic burden of PD within the U.S. and European nations has recently reached billions of dollars (4). Several studies have reported associations between PD and several systemic diseases, including inflammatory bowel disease, diabetes mellitus, cardiovascular diseases, cognitive decline and Alzheimer’s disease, chronic obstructive pulmonary disease, and certain cancers (3, 6–23). These studies propose mechanisms by which inflammation and microbial dysbiosis in the oral cavity can lead to these systemic conditions, but determining directionality is still a matter of investigation. Nonetheless, the economic and health consequences of PD warrant further investigation of the cellular and molecular mechanisms behind PD induced inflammation which can provide insights into preventative care for PD and, possibly, reduce the risk of associated systemic diseases.

The pathogenesis of PD typically begins with the formation of dental plaque biofilm on the surface of teeth which then infiltrate the periodontal pockets surrounding teeth (24). Evidence suggests that microbial species associated with PD severity, such as *Porphyromonas gingivalis*, *Treponema denticola*, and *Tanneralla forsythia*, colonize established dental plaque leading to a microbial shift from higher levels of symbiotic bacteria to higher levels of pathogenic bacteria within the plaque. Previous studies have identified bacterial species that may serve as “bridging species” which promote the adhesion of PD associated pathogens (e.g., *P. gingivalis*) and other secondary colonizers to existing plaque (3, 25–27). Specifically, *Streptococcus* spp. and *Actinomyces* spp. have been found to serve as early colonizers which can promote the adhesion of intermediate colonizers, including *Fusobacterium* spp., which in turn promote the adhesion of PD associated pathogens (26, 28, 29). As bacteria colonize teeth at and below the gingival margin, an inflammatory response is initiated as leukocytes, including neutrophils and T cells, begin to infiltrate and eliminate colonizing bacteria. However, pathogenic bacteria produce virulence factors that allow them to evade destruction via several mechanisms, including degrading cytokines, producing capsules, residing intracellularly within immune cells, and releasing toxins that directly damage leukocytes (3, 30, 31). Interactions between pathogenic bacteria and the host immune system during PD can lead to chronic inflammation which, if left untreated, can lead to the observed periodontal pathology—soft tissue and alveolar bone destruction (25, 32, 33). Although the presence of PD pathogens is typically a prerequisite for PD pathogenesis, the soft tissue and alveolar bone loss observed is largely driven by the host immune response. Of note, a landmark study by Loe et al. in 1986 demonstrated how a group of individuals with uniformly poor oral hygiene separated into groups of rapid, moderate, and no progression of PD (34). This study and others have described well the differences in disease progression among patients, but there is still a gap in our understanding on how the host immune response can leave some patients severely afflicted due to poor oral hygiene while others experience little to no disease progression. Several pharmacologic agents have been explored to modulate the immune response for the treatment of periodontitis (sub-antimicrobial-dose doxycycline (SSD), nonsteroidal anti-inflammatory drugs (NSAIDs), and bisphosphonates). In practice, many dentists rely primarily on the physical removal of calculus and plaque from the tooth surface using stainless steel instruments (inexpensive), while costly medications tend to offer statistically significant but clinically modest benefits. For example, a review by Preshaw et al. notes that adjunctive SSD resulted in clinical attachment gains that were 19% greater than with a placebo (2.62 mm versus 2.20 mm) (35). Other agents such as NSAIDs and bisphosphonates are limited by long-term side effects or risk of complications like medication-related osteonecrosis of the jaw (MRONJ). Additionally, a systematic review by Haffajee in 2003 concluded that systemic antibiotics used as monotherapy (without instrumentation) are ineffective, reinforcing the clinical assumption that mechanical debridement is indispensable (36). Few host-modulating agents for periodontitis exist, and non-surgical techniques have remained largely unchanged for decades. Thus, identifying a new immunotherapy would represent a meaningful step forward.

CD4^+^ helper T cells and CD8^+^ cytotoxic T cells are both are implicated in PD (37–40). Th1 cells secrete proinflammatory cytokines such as interferon-gamma (IFNγ) and tumor necrosis factor (TNF) and initiate a type I cell mediated response, Th2 cells secrete cytokines including interleukin 4 (IL4) and IL13 to enable a type II humoral response, and Th17 cells secret IL17A and support a type III response. Tregs reduce inflammation by secreting the anti-inflammatory cytokine IL10 and the pleiotropic transforming growth factor-beta (TGFβ) which can inhibit inflammation but also promote Th17 differentiation. IL-17A and IL-6 are associated with PD pathogenesis, and salivary levels of both are significantly higher in patients with PD compared to healthy individuals, with levels increasing as the disease progresses (41). IL-6 and IL-17A may also play critical roles in promoting alveolar bone resorption and tissue destruction by inducing Receptor Activator of Nuclear Factor-κB Ligand (RANKL) expression (42, 43). RANKL is a cytokine that is primarily secreted by activated T cells and B cells during PD, and it promotes the differentiation and activation of osteoclasts leading to alveolar bone resorption (44–46).

In 2016, Dutzan et al. characterized the percentages of immune cell types found in healthy gingiva and buccal mucosa using flow cytometry (40). During homeostasis, T cells are the dominant immune cell population in healthy gingiva and buccal mucosa with CD4^+^ T cells being more numerous than CD8^+^ T cells and 10-15% of the CD4^+^ T cells within healthy gingiva being Foxp3^+^ Tregs (40). Additionally, the investigators found that 80% and 50% of CD4^+^ and CD8^+^ T cells, respectively, in healthy gingiva were memory cells with the majority being resident effector memory cells for both subsets. During periodontal inflammation, the total number of T cells increases, and CD4^+^ cells are the dominant source of IL-17 (40, 47). Th1, Th2, and Th17 cells have been shown to produce pro-inflammatory cytokines that can activate and recruit dendritic cells, neutrophils, and B cells during PD leading to their increased levels observed in PD gingiva (38, 40, 48, 49). A study conducted by Li et al. in 2020 utilized gene expression data and the CIBERSORT software to identify immune cell types from 133 healthy human periodontal tissues and 210 chronic PD tissues (48). Their analysis identified that PD tissues had increased levels of activated memory CD4^+^ cells compared to healthy tissues while seeing a concurrent decrease in resting memory CD4^+^ T cells, and Tregs, and CD8^+^ T cells. However, their CIBERSORT analysis determined that total T cell counts were lower in PD tissues, contrary to the flow cytometry data analyzed by Dutzan et al. Despite some discrepancies, these recent studies show that there is a shift in T cell activity during PD pathogenesis leading to increased inflammation.

CD5 is glycoprotein found on the surface of T cells and a subset of mature B cells, B1a cells. Of note, circulating CD5^+^ B cells have been found to be enriched in periodontitis patients (50). Though the ligands and signaling pathway of CD5 are still being investigated, recent work has provided insight into the role CD5 plays in regulating T cell activity. Previous studies have shown that CD5 signaling attenuates T cell receptor (TCR) signaling during thymic development and that levels of CD5 on T cells may serve as a marker of TCR self-affinity (51, 52). In the periphery, CD5 has been shown to inhibit T cell activation while also promoting Treg differentiation and influencing memory formation (53, 54). The recruitment of several cytoplasmic proteins to the cytoplasmic tail of CD5 including Ras GTPase-activating protein (rasGAP), phosphatidylinositol 3-kinase (PI3K), casein kinase 2 (CK2), and CBL has been suggested to mediate TCR attenuation and influence Treg and memory formation (55–58). Recent studies investigating the role of CD5 in regulating T cell activity in response to disease, including responses to sepsis and cancer, indicate that CD5 may serve as an inhibitory or co-stimulatory receptor depending on the physiological context (59–63). Though there is a growing repertoire of studies investigating CD5 as a potential immunotherapy target for cancer, there are currently no studies investigating the role CD5 plays in T cell activity and chronic inflammation during PD. Thus, we sought to determine whether removal of CD5 from T cells would exacerbate or inhibit their activity in the context of PD.

In the present study, the role of CD5 in regulating T cell activation and differentiation is investigated in the context of PD using an *in vitro* model to examine the effects of *P. gingivalis* LPS on oral epithelial and immune cells. To mimic the inflammatory response that occurs during PD, lipopolysaccharide (LPS) from *P. gingivalis* was used to stimulate mouse mucosal and gingival epithelia to induce the secretion of inflammatory cytokines into the cell media akin to epithelia responding to bacterial antigens during PD. This model was inspired by previous models that have used *P. gingivalis* antigens to stimulate human oral epithelial cell lines to produce cytokines, as well as a model that used LPS to stimulate a human lung epithelial cell line prior to using the cell supernatant to stimulate leukocytes (64–67). In our model, LPS from *E. coli* was used as a standard to compare the immunogenicity of LPS from *P. gingivalis* during assays. The epithelial supernatant was then added to mouse splenocyte suspensions where plate-bound anti-mouse CD3ϵ antibody and soluble anti-mouse CD28 antibody were used to provide the primary and secondary signals to fully activate the splenic T cells. Our results indicate that removal of CD5 on T cells in this *in vitro* model enhances T cell activation and promotes effector differentiation, CD8+ T cell memory formation, and alters cytokine production. Thus, CD5 may serve as a novel immunotherapy target to modulate inflammation during PD.

## 2 Methods

### 2.1 Mice

C57BL/6 CD5 knockout (KO) and Wild type (WT) mice were bred and housed in pathogen free conditions. All mice used in these experiments were 8–12 weeks old. All use of laboratory animals was done with approval of the Animal Care and Use Committee (IACUC protocol #24-0823) at Brigham Young University.

### 2.2 Mouse gingival and oral mucosal epithelial cell culturing

Mouse gingival (C57–6202 Cell Biologics) and oral mucosal epithelial cells (C57–6234 Cell Biologics) were isolated from C57BL/6 mouse gingival and mucosal tissue respectively and were used at passage 5 or lower and were cultured in 10mL of complete epithelial cell medium (M6621, Cell Biologics) in separate tissue culture flasks (25-209, GenClone) for 48 hours or until 70-80% confluency. The cells were then detached from the flask by removing the media, washing the plate with 1x phosphate-buffered saline (PBS), adding trypsin 0.05% EDTA, and incubating at 37 °C for 5 minutes. The detached cells were collected in 15mL conical tubes and centrifuged at 150 g for 5 minutes to pellet the cells. The cells were resuspended in complete epithelial cell medium, and cell concentration was determined using an Olympus R1 automated cell counter. 400,000 mouse gingival and oral mucosal epithelial cells were seeded, separately, into sterile 6-well plates at a final volume of 4mL of Complete Epi media and cultured for 24 hours. The media in each well was replaced with 4mL R10 media, and the epithelial cells were stimulated to release inflammatory cytokines by adding either *Porphyromonas gingivalis* lipopolysaccharide (PG-LPS) (tlrl-pglps, InvivoGen) or *Escherichia coli* (EC-LPS) (tlrl-b5lps, InvivoGen) and incubating for 24 hours. The supernatant from stimulated epithelial cells was then used to stimulate mouse splenocytes or isolated CD4^+^ T cells during functional assays.

### 2.3 Splenic T cell activation and flow cytometric analysis of T cell activation and differentiation

Spleens from WT and CD5 knockout mice were harvested and placed into 6-well plates filled with R10 media. Each biological replicate represents one wildtype or CD5KO mouse. The spleens were mashed using the plunger of a 3mL syringe through cell strainers to generate single cell suspension of splenocytes. The cell suspensions were treated with ammonium-chloride-potassium (ACK) lysis buffer for less than 1 minute to lyse red blood cells, and the reaction was quenched using R10 media. Splenocytes were pelleted, resuspended, and counted an Olympus R1 automated cell counter. 200,000 splenocytes contained in 100μL R10 media were then seeded into 96-well plates coated with anti-mouse CD3e antibody (precoated with 200μL of 5μg/mL antibody PBS solution). Soluble CD28 antibody (2μg/mL) was added to all wells except for unstimulated, and 100μL of stimulated epithelial supernatant were added to designated wells. “Unstimulated” and “CD3/28 No Supernatant” wells received 100μL R10 media instead of epithelial supernatant. The cells were allowed to incubate at 37 °C and 5% CO2. For wells treated with polymyxin B, the drug was added to each well at a final concentration of 100U/mL diluted in R10 media. For wells that received only LPS (no epithelial supernatant), PG- or EC-LPS were added directly to the splenocytes at final concentrations of 10μg/mL and 1μg/mL, respectively, to determine what role residual LPS played in altering epithelial and immune cell function separate from inflammatory mediators that may be included in the epithelial supernatant. After the incubation (1–5 days), the splenocytes were pelleted and resuspended in blocking solution containing CD16/32 antibody (1:100 dilution, Biolegend) and LIVE/DEAD Violet dye (1:1000 dilution, L34955, Thermofisher) and incubated at room temperature for 10 minutes. Surface staining solution was then added to the cells and incubated at 4 °C protected from light for 20 minutes using the following antibodies: CD4-APCeFluor780 (47-0041-82, Invitrogen), CD8a-PeCy5.5 (35-0081-82, Thermofisher), CD44-APC (103012, Biolegend), CD62L-BV605 (104438, Biolegend), CCR7-AlexaFluor700 (56-1971-82, Thermofisher), CD69-FITC (104506, Biolegend), and CD25-APC (102012, Biolegend). Samples were then fixed with Foxp3/Transcription Factor Staining Fixation Buffer (00-5523-00, Thermofisher) for at least 30 minutes at 4 °C. The cells were then intracellularly stained for Foxp3 or IL17A for 20 minutes at 4 °C protected from light using Foxp3-PE (126404, Biolegend) and IL17A-APC (506916, Biolegend) antibodies diluted in Foxp3/Transcription Factor Permeabilization Buffer (1:100 dilution). Samples were pelleted and resuspended in 60μL PBS and analyzed using the Cytoflex S Flow Cytometer. Flow cytometry data analysis was performed using FlowJo software (v.10). Statistical analysis was performed using GraphPad Prism version 11.0.1. Two-way ANOVA with multiple comparisons with Sidak correction with a single pooled variance was performed to determine statistical significance between wildtype and CD5KO in each stimulation type, along with determining the variance in the means of cell subset percentages between wildtype and CD5KO due to genotype with associated p-values.

### 2.4 Cytometric bead array analysis

Supernatant was collected from splenocyte sample wells (described in Methods section Splenic T cell activation and Flow cytometric analysis of T cell activation and differentiation) on days 3 and 5 post stimulation and stored at -20 °C until further analysis using the BD™ Cytometric Bead Array (CBA) Mouse Th1/Th2/Th17 CBA Kit (560485, BD Biosciences) following the manufacturer’s protocol. The cytometric beads were analyzed using the Cytoflex S Flow Cytometer (Beckman Coulter), and FlowJo software (v.10) was used to measure the median fluorescence intensity (MFI) of the detection reagent for each cytokine. A standard curve was generated using the cytokine standards supplied by the kit, and a non-linear fit line was used to determine the concentrations of cytokines in the test samples. Statistical analysis was performed using GraphPad Prism version 11.0.1. Two-way ANOVA and multiple comparisons with Sidak correction with a single pooled variance was performed to determine statistical significance between wildtype and CD5KO in each stimulation type, along with determining the variance in the means of supernatant cytokine concentrations between wildtype and CD5KO due to genotype with associated p-values.

### 2.5 Real-time quantitative PCR

Spleens from WT and CD5KO female mice aged 9–12 weeks were used for RT-qPCR experiments, and splenic CD4^+^ T cells were isolated using the StemCell EasySep Mouse CD4^+^ T cell isolation kit (Catalog # 19852). For T cell stimulation, 200,000 CD4^+^ T cells in 500μL R10 were seeded in 24-well plates coated with anti-mouse CD3e antibody (precoated with 400μL of 5μg/mL antibody PBS solution) (14-0031-86, Thermofisher). Soluble anti-mouse CD28 antibody (final concentration = 2 μg/mL) (16-0281-86, Thermofisher) and 500μL LPS-stimulated epithelial supernatant were also added to stimulated wells (epithelial supernatant from *Mouse gingival and oral mucosal epithelial cell culturing*). 200,000 unstimulated T cells were cultured in 24-well plates containing 1mL R10. Both stimulated and unstimulated T cells were incubated for 3 days within an incubator set at 37 °C and 5% CO2. Total RNA was isolated from WT and CD5KO stimulated and unstimulated splenic CD4^+^ T cells using the Quick-RNA™ Miniprep Kit (Catalog # R1054). Comparative RT-qPCR was performed on three biological replicates of stimulated and unstimulated WT and CD5KO CD4+ T cells, with three technical replicates for each biological sample, using primers for genes Csf1, Rankl, and β-actin as the housekeeping gene (sequences below). SYBR Green was used as the fluorescent dye. Ct values for Csf1 and Rankl in both CD5KO and wildtype samples were normalized to the Ct value of β-actin from the same samples. Fold differences between wildtype and CD5KO samples in Csf1 and Rankl transcription were then calculated by comparing the CD5KO sample Ct values to the Ct values of wildtype samples. Fold changes between unstimulated samples and the various stimulus types was also calculated by comparing Ct values of stimulated wildtype and CD5KO to their respective unstimulated samples (e.g., unstimulated wildtype vs. WT GPG).

Csf1 Forward Sequence: GCCTCCTGTTCTACAAGTGGAAG.

Csf1 Reverse Sequence: ACTGGCAGTTCCACCTGTCTGT.

Rankl Forward Sequence: GTGAAGACACACTACCTGACTCC.

Rankl Reverse Sequence: GCCACATCCAACCATGAGCCTT.

β-actin Forward Sequence: CATTGCTGACAGGATGCAGAAGG.

β-actin Forward Sequence: TGCTGGAAGGTGGACAGTGAGG.

### 2.6 LPS quantification

The Pierce Chromogenic Endotoxin Quant Kit (A39552, ThermoFisher) was used according to the manufacturer’s instructions to quantify residual LPS levels in epithelial supernatants. Briefly, 50μL of each supernatant was transferred to a clear, flat bottom 96-well plate. Sample wells, standards, and blanks were prepared in duplicate. 50μL of Amebocyte Lysate Reagent was added to each well and allowed to incubate at 37C for 28 minutes, after which 100μL of Chromogenic Substrate was added to each well and allowed to incubate at 37C for 6 minutes. The reaction was stopped using 25% acetic acid, and the optical density of each well was analyzed at 405nm. Concentrations of LPS were determined by formulating a standard curve using the provided standards. 1EU is approximately 0.1-0.2ng of LPS.

## 3 Results

### 3.1 Removal of CD5 from mouse T cells enhances CD8^+^ T cell activation in an *in vitro* model used to investigate the effects of *P. gingivalis* LPS on oral epithelial cells

**Figure 1:**
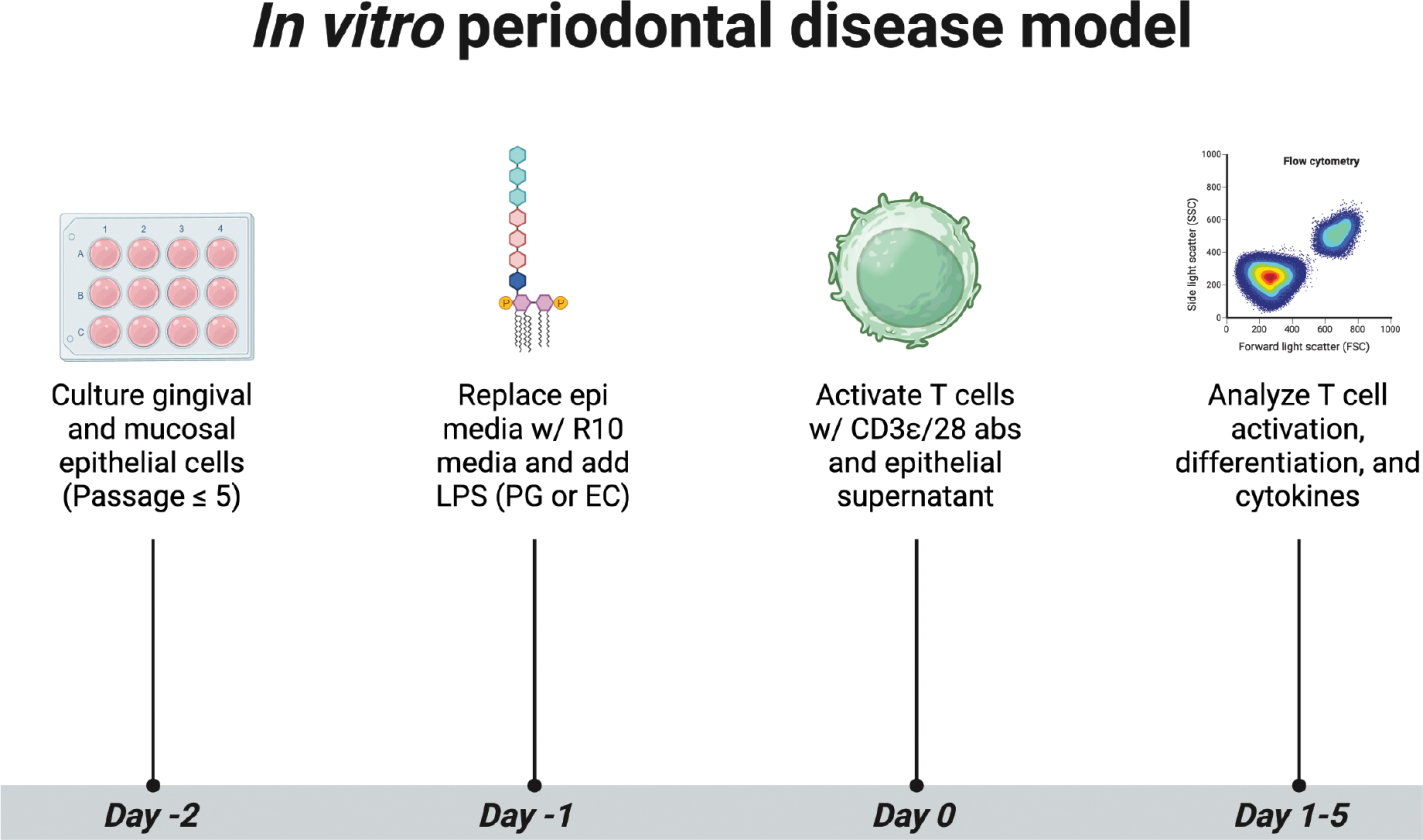
Graphical representation of the *in vitro* model used to investigate the effects of *P. gingivalis* LPS on oral epithelial and immune cells. Mouse gingival and oral mucosal epithelial cells (passage 5 or lower) were cultured in 6-well plates (400,000 cells) containing 4mL of Complete Epithelia Media for 24 hours (Day -2). Then, the media was replaced with fresh R10 media and LPS from *P. gingivalis* or *E. coli* was added to stimulate the epithelial cells for 24 hours (Day -1). The supernatant from the epithelial cells was then used to stimulate mouse splenocytes or isolated CD4+ T cells during functional assays, in addition to CD3ϵ/28 antibody stimulation (Day 0). T cells were then analyzed to assess activation, differentiation and cytokine production after 1–5 days of stimulation. Created in BioRender. Moreno, C. (2026).

**Figure 2:**
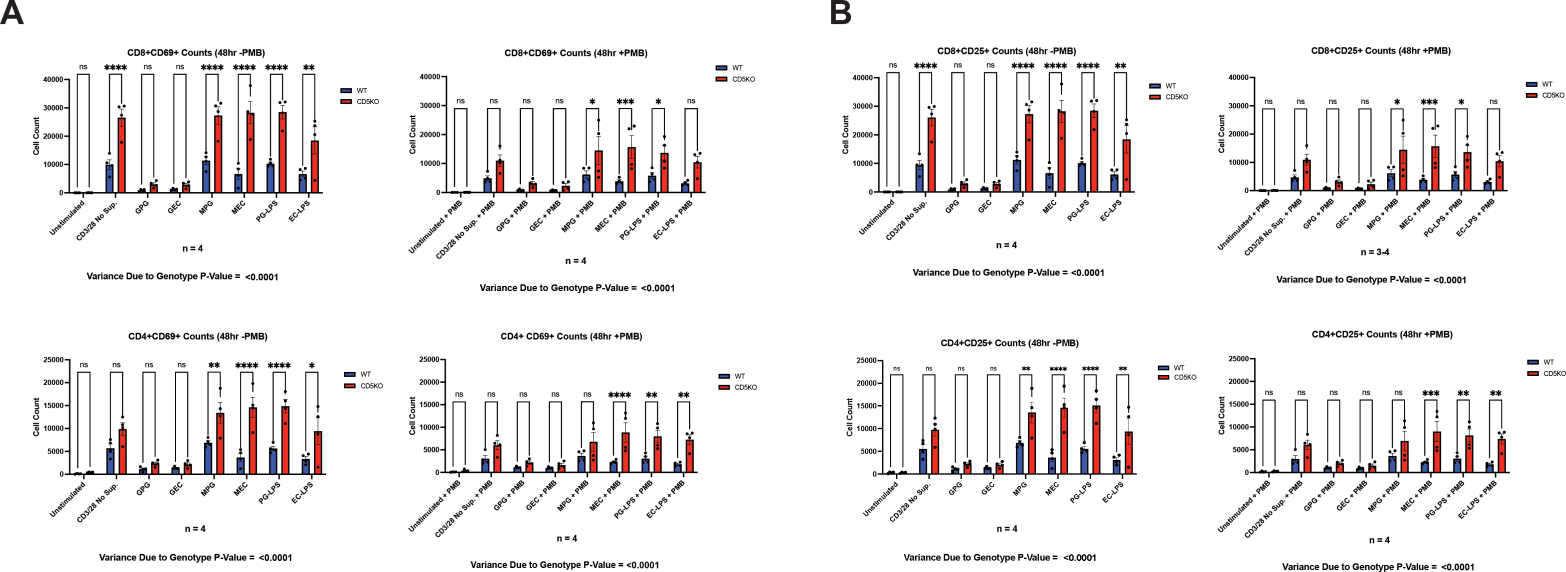
Cell counts of CD69+ wildtype and CD5 knockout T cells post 48-hours stimulation. After stimulation for 48 hours, **(A)** CD69 and **(B)** CD25+ expression on CD4+ and CD8+ T cells were assessed via flow cytometry. Ordinary two-way ANOVA with Sidak’s multiple comparisons test to determine statistical significance. GPG, Gingival epithelial cell supernatant treated with P. gingivalis LPS. GEC, Gingival epithelial cell supernatant treated with E. coli LPS. MPG, Mucosal epithelial cell supernatant treated with P. gingivalis LPS. MEC, Mucosal epithelial cell supernatant treated with E. coli LPS. PG-LPS, P. gingivalis LPS. EC-LPS, E. coli LPS. PMB, Polymyxin B treatment (100 U/mL). *p-value < 0.05, **p-value < 0.005, ***p-value < 0.0002, ****p-value < 0.0001. ns means not significant.

Lipopolysaccharide (LPS) from *P. gingivalis* was used to stimulate mouse mucosal and gingival epithelia to induce the secretion of inflammatory cytokines into the cell media akin to epithelia responding to bacterial antigens during PD and *E. coli* LPS was used as a control (**Figure 1**). The cell media containing secreted cytokines and LPS was added to mouse splenocyte suspensions where plate-bound anti-mouse CD3ϵ antibody and soluble anti-mouse CD28 antibody were used to provide the primary and secondary signals to fully activate the splenic T cells (**Figure 1**). At 6, 12, 24, and 48 hours post stimulation with CD3ϵ/CD28 antibodies and supernatant from gingival or mucosal epithelia stimulated with PG-LPS or EC-LPS (**Figure 2**; **Supplementary Figures 1**–**3**). Since residual LPS was observed in epithelial supernatants (**Supplementary Figure 4**), separate wells with the same stimulation types were treated with polymyxin B to block LPS and help determine if differences in activation kinetics were due in part to residual LPS in the media. Importantly, we observed relatively low levels of residual LPS in the epithelial supernatants with about 1.5EU/mL of LPS in all four epithelial supernatant types (**Supplementary Figure 4**). 1EU is approximately 0.1-0.2ng of LPS. LPS only control wells were also included. CD5 knockout CD8^+^ T cells had an increase in CD25^+^and CD69^+^ activated cells compared to wildtype across several stimulation types by 48 hours of stimulation (**Figure 2**). There was a statistically significant increase in CD8^+^CD69^+^ and CD8^+^CD25^+^ CD5 knockout T cell counts when activated with only CD3ϵ/CD28 antibodies, as well as supernatant from mucosal epithelial cells but not from gingival epithelial cells (**Figure 2**). Additionally, we observed marked increases in CD4^+^CD69^+^ and CD4^+^CD25^+^ counts when cultured with supernatant from stimulated mucosal epithelial cells (**Figure 2**). Increases in activated T cell counts among CD5KO T cells were also observed in LPS only wells. When T cells were treated with polymyxin B to block LPS, activated T cell counts across all stimulation types were notably lower compared to non-treated T cells (68). This suggests that polymyxin B treatment may, itself, have a direct impact on T cell activation (68). Importantly, we did not observe a dramatic change in T cell activation differences between wildtype and CD5KO across stimulation types and time points due to polymyxin B treatment that would indicate residual LPS has a dramatic role in activating T cells in our model. The observed increases in activation after stimulation among CD5 knockout CD8^+^ T cells compared to wildtype is congruent with previous studies (69, 70). Thus, removal of CD5 is confirmed to increase CD4^+^ and CD8^+^ T cell activation in this *in vitro* model.

### 3.2 Removal of CD5 alters effector/memory differentiation among CD4^+^ and CD8^+^ T cells in *in* an *in vitro oral epithelial cells model for P. gingivalis* LPS effect

**Figure 3:**
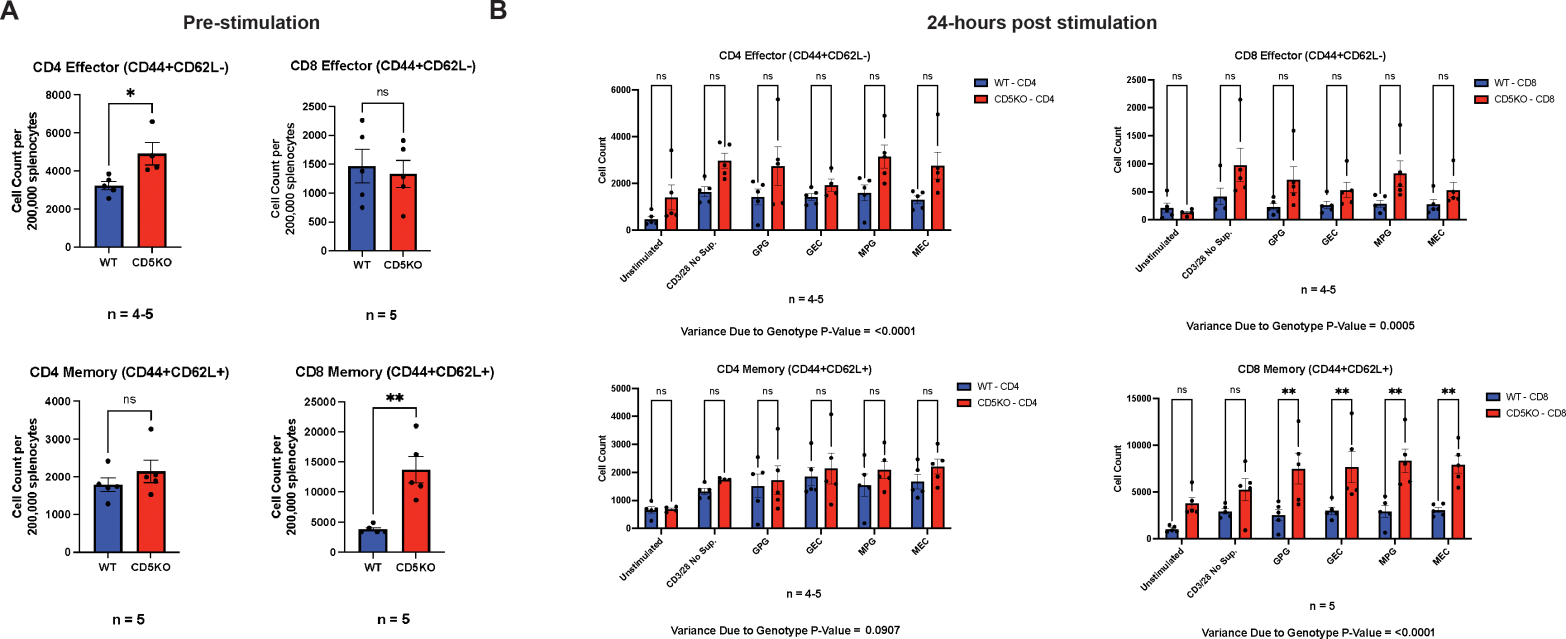
Cell counts of effector and memory T cell subsets before and after 24 hours stimulation. **(A)** Cell counts of Effector (CD44+CD62L-) and Memory (CD44+CD62L+) CD4+ and CD8+ T cells before stimulation. Ordinary two-way ANOVA with Sidak’s multiple comparisons test to determine statistical significance. **(B)** Cell counts of Effector and Memory T cells after 24-hours stimulation. GPG, Gingival epithelial cell supernatant treated with *P. gingivalis* LPS. GEC, Gingival epithelial cell supernatant treated with *E. coli* LPS. MPG, Mucosal epithelial cell supernatant treated with *P. gingivalis* LPS. MEC, Mucosal epithelial cell supernatant treated with *E. coli* LPS. *p-value < 0.05, **p-value < 0.005. ns means not significant.

**Figure 4:**
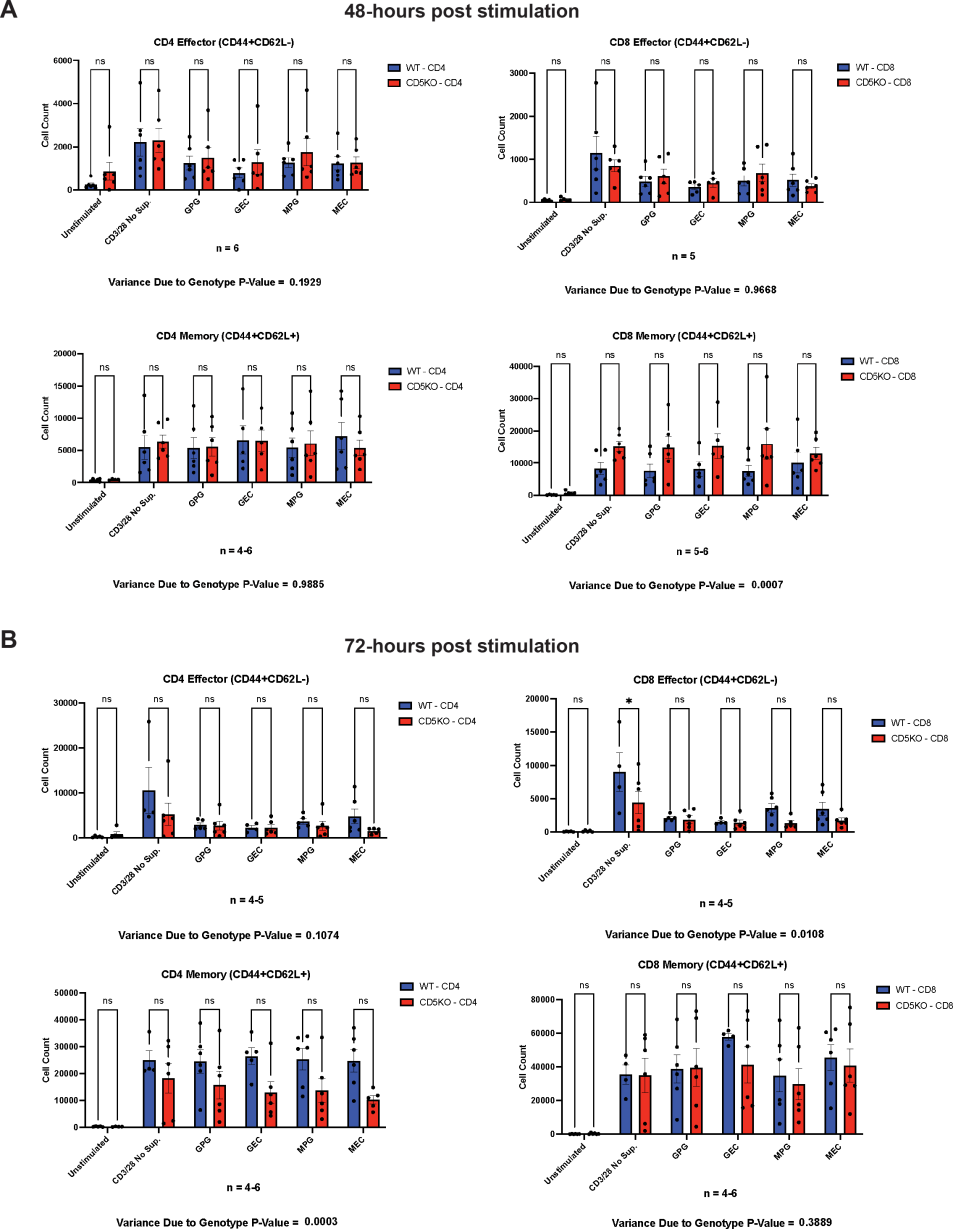
Cell counts of effector and memory T cell subsets before and after stimulation (48 and 72 hours). **(A)** Cell counts of Effector (CD44+CD62L-) and Memory (CD44+CD62L+) CD4+ and CD8+ T cells after 48 hours of stimulation. Ordinary two-way ANOVA with Sidak’s multiple comparisons test to determine statistical significance. **(B)** Cell counts of Effector and Memory T cells after 120-hours stimulation. Ordinary two-way ANOVA with Sidak’s multiple comparisons test to determine statistical significance. GPG = Gingival epithelial cell supernatant treated with P. gingivalis LPS. GEC, Gingival epithelial cell supernatant treated with E. coli LPS. MPG, Mucosal epithelial cell supernatant treated with P. gingivalis LPS. MEC, Mucosal epithelial cell supernatant treated with E. coli LPS. *p-value < 0.05. ns means not significant.

CD4^+^ and CD8^+^ effector and memory cell counts were measured among wildtype and CD5 knockout splenocytes prior to stimulation, and there was a statistically significant increase in CD4^+^ effector cells and CD8^+^ memory cells among CD5 knockout splenocytes compared to wildtype (**Figure 3A**). After 24 hours of stimulation, CD5 knockout continued to have increased CD8^+^ memory cells across all epithelial supernatant stimulation types compared to wildtype, but there were no differences between CD5KO and wildtype when stimulated with only CD3ϵ/28 antibody (**Figure 3B**). 48 hours after activation, there were no longer any statistically significant differences observed between wildtype and CD5KO across all of the stimulation types, but ordinary two-way ANOVA determined that variance due to genotype was statistically significant in CD8 memory cell counts (p-value = 0.0007) (**Figure 4A**). After 72 hours of activation, the only direct comparison that was statistically different was the increase in effector T cell counts observed among wildtype CD8^+^ T cells when stimulated with only CD3ϵ/28 antibodies (**Figure 4B**). However, ordinary two-way ANOVA again determined that variance due to genotype was statistically significant among CD8^+^ effector (p-value = 0.0108) and CD4^+^ memory (p-value = 0.0003) cell counts. These findings suggest that removal of CD5 affects the kinetics of memory T cell differentiation in this *in vitro* model as differences were observed at 24 hours but no longer at 48 and 72 hours due to WT CD8+ memory counts catching up with CD5KO by the later time points. Additionally, removal of CD5 possibly affects the kinetics of CD4^+^ and CD8^+^ effector differentiation, though this effect is weak as indicated by the lack of statistically significant differences as determined by multiple comparisons except for one instance (CD8 Effector CD3/28 No Sup. at 72 hours) but supported by 2-way ANOVA determining variance due to genotype was statistically significant in several instances as mentioned above.

### 3.3 CD5 deletion does not alter Treg cell differentiation in this *in vitro* model

Prior to stimulation, there was no difference in Treg (CD4^+^CD25^+^Foxp3^+^) counts between wildtype and CD5 knockout splenocytes (**Supplementary Figure 5A**). After stimulation 1 to 3 days, there were still no statistically significant differences between wildtype and CD5 knockout Treg counts across the stimulation types (**Supplementary Figures 5B–D**). Ordinary two-way analysis determined that variance due to genotype was statistically significant among Treg counts at 72-hours post stimulation.

### 3.4 CD5 regulates Th17 differentiation but not in this *in vitro* model

**Figure 5:**
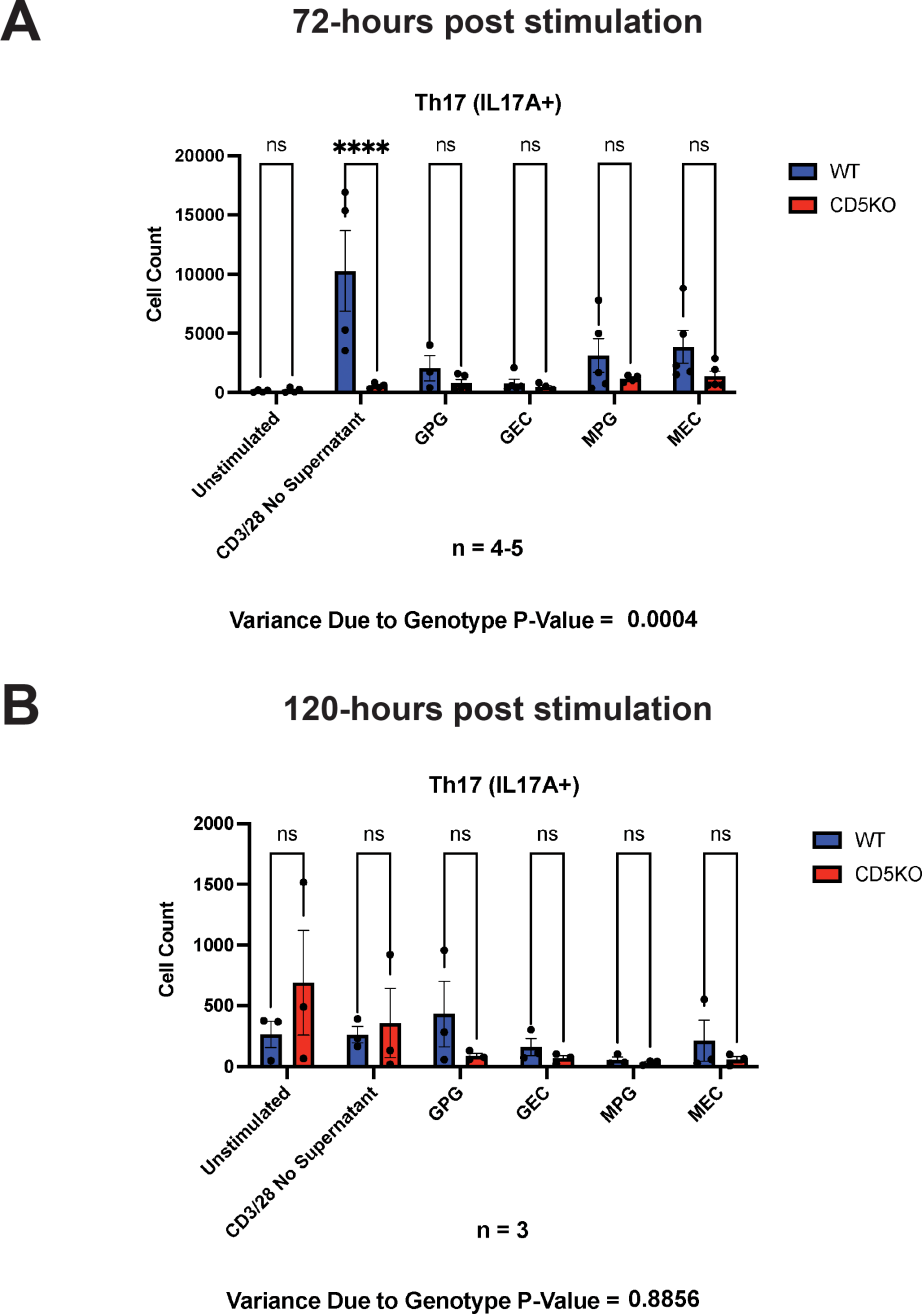
Comparison of Th17 counts between wildtype and CD5 knockout CD4+ T cells. Cell counts of Th17 cells (CD4+IL17A+) after **(A)** 72 hours and **(B)** 120 hours of stimulation. Ordinary two-way ANOVA with Sidak’s multiple comparisons test to determine statistical significance. GPG, Gingival epithelial cell supernatant treated with *P. gingivalis* LPS. GEC, Gingival epithelial cell supernatant treated with *E. coli* LPS. MPG, Mucosal epithelial cell supernatant treated with *P. gingivalis* LPS. MEC, Mucosal epithelial cell supernatant treated with *E. coli* LPS. ****p-value < 0.0001. ns means not significant.

Th17 cells were quantified at 72- and 120-hours post stimulation to determine if removal of CD5 affected Th17 differentiation. After 72 hours of activation, there was an increase in Th17 counts among wildtype CD4^+^ cells compared to CD5 knockout when stimulated with only CD3ϵ/28 antibodies (**Figure 5A**). Ordinary two-way analysis determined that variance due to genotype was statistically significant among Th17 cell counts at 72-hours post stimulation (p-value = 0.0004). We speculate that supernatants from gingival epithelial cells stimulated by PG-LPS or EC-LPS may be inhibiting Th17 counts compared to stimulation without supernatant, indicating an inhibitory effect by these epithelial cells on Th17 differentiation, though this effect has not been established. By day 5 (120 hours), there were no differences in Th17 counts between wildtype and CD5 knockout (**Figure 5B**).

### 3.5 Removal of CD5 alters T cell cytokine production in this *in vitro* model

**Figure 6:**
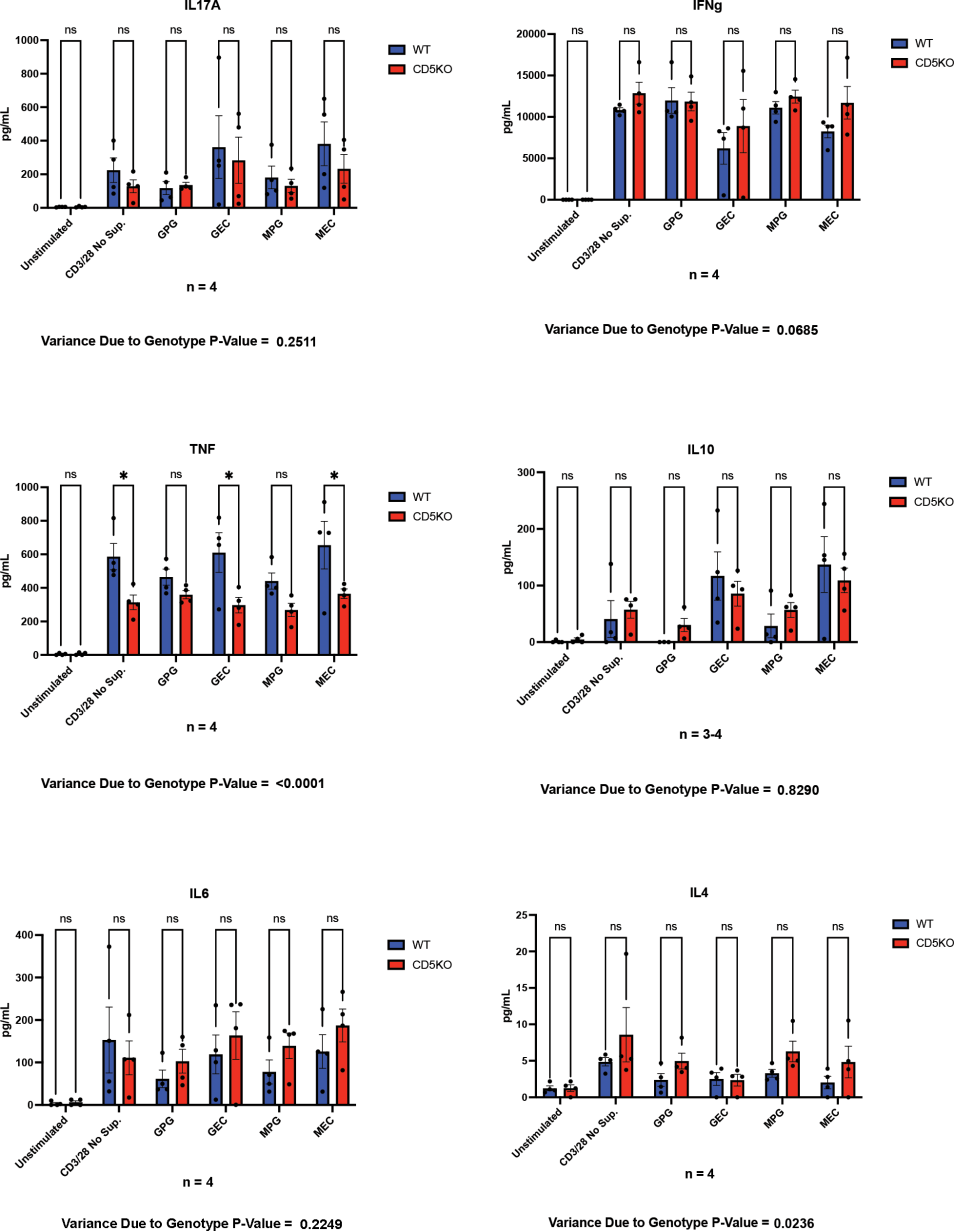
Levels of Th1/Th2/Th17 associated cytokines produced by wildtype and CD5 knockout T cells in this *in vitro* model after 72 hours of stimulation. Concentrations of cytokines secreted by Th1, Th2, and Th17 cells in cell supernatants after stimulation for 72 hours was measured using a cytokine bead array. Ordinary two-way ANOVA with Sidak’s multiple comparisons test to determine statistical significance. GPG, Gingival epithelial cell supernatant treated with *P. gingivalis* LPS. GEC, Gingival epithelial cell supernatant treated with *E. coli* LPS. MPG, Mucosal epithelial cell supernatant treated with *P. gingivalis* LPS. MEC, Mucosal epithelial cell supernatant treated with *E. coli* LPS. *p-value < 0.05. ns means not significant.

After 72 hours of stimulation, cytokines associated with Th1, Th2, and Th17 subsets in the cell supernatant were measured using the BD™ Cytometric Bead Array (CBA) Mouse Th1/Th2/Th17 CBA Kit (560485, BD Biosciences). Tumor necrosis factor (TNF) was increased among wildtype cells compared to CD5 knockout in the “CD3/28 No Supernatant” and EC-LPS stimulated epithelial supernatant wells, with no statistically significant differences in the PG-LPS stimulated epithelial supernatant wells (**Figure 6**). Ordinary two-way analysis determined that variance due to genotype was statistically significant among TNF (p-value < 0.0001) and IL4 (p-value = 0.0236) cytokine levels. The only differences observed between wildtype and CD5 knockout cytokine levels on 120-horus post stimulation were an increase in TNF levels among wildtype cells when stimulated with mucosal epithelial cell supernatant stimulated with EC-LPS and an increase in IL4 levels among CD5KO cells when stimulated with only CD3ϵ/28 antibodies (**Supplementary Figure 6**). Ordinary two-way analysis determined that variance due to genotype was statistically significant among TNF (p-value = 0.0012), IFNγ (p-value = 0.0028), and IL4 (p-value = 0.0206) levels.

**Figure 7:**
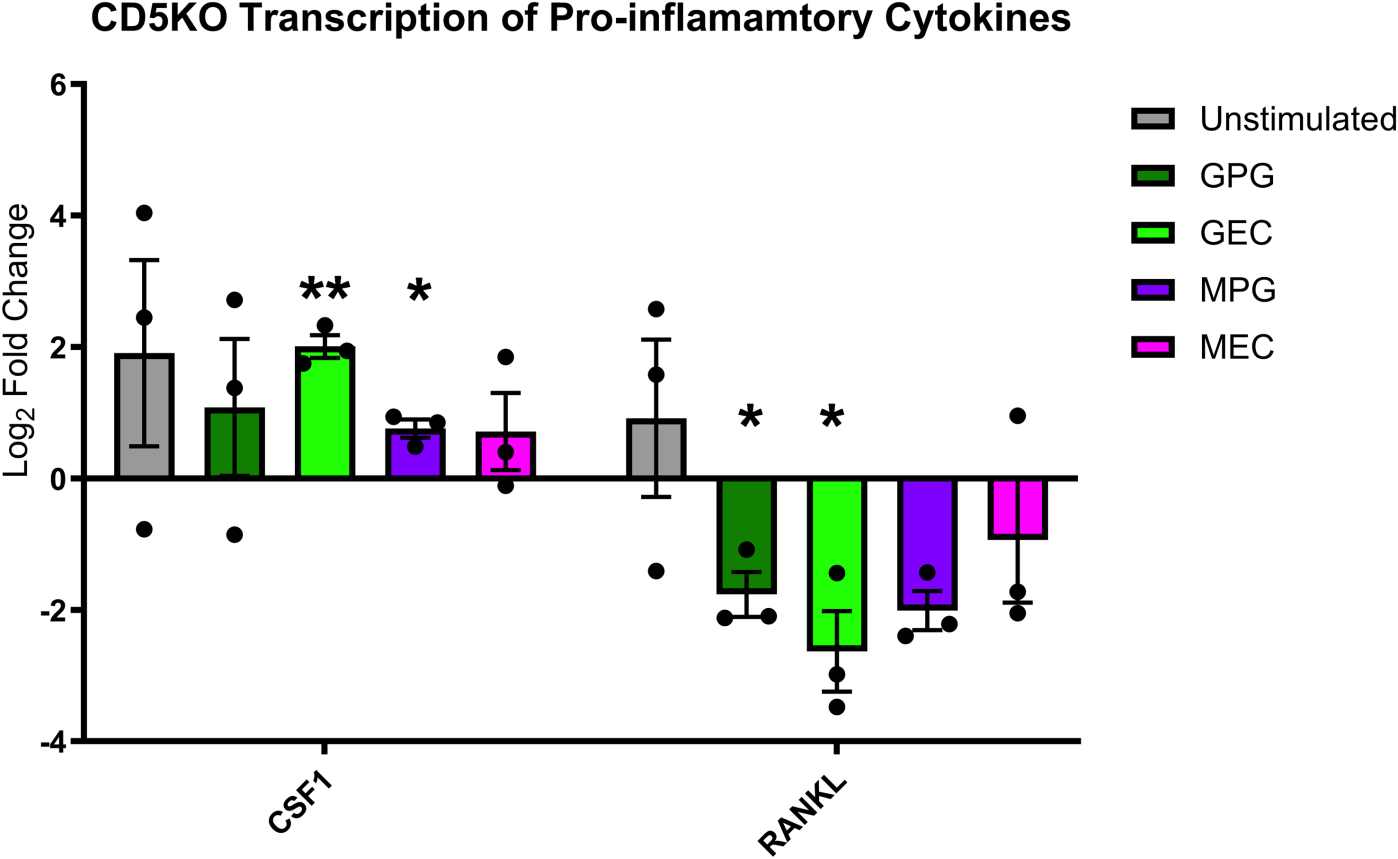
CD5KO CD4+ T cell mRNA transcription of pro-inflammatory cytokines compared to WT CD4+ T cells. WT and CD5KO CD4+ T cells (n=3) were stimulated for 72 hours with plate bound anti-mouse CD3ϵ antibody, soluble anti-mouse CD28 antibody, and supernatant from PG- or EC-LPS-stimulated mouse gingival or oral mucosal epithelial cells. Total RNA was isolated from the stimulated T cells and converted into cDNA prior to RT-qPCR analysis using Power SYBR Green Master Mix. Unstimulated controls were mRNA samples isolated from CD4+ T cells immediately after cell isolation from the spleens of WT and CD5KO mice. Log_2_ fold change of CD5KO mRNA transcription is indicated on the y-axis. The x-axis indicates the gene of interest. The color of the bars indicates the stimulus type, GPG, gingival epithelia + PG-LPS supernatant, GEC, gingival epithelia + EC-LPS supernatant, MPG, oral mucosal epithelia + PG-LPS supernatant, MEC, oral mucosal epithelia + EC-LPS supernatant. The log2 fold change values were compared to a null hypothetical mean of 0 using one-sample t-tests (*p-value < 0.05, **p-value < 0.005).

CSF1 and receptor activator of RANKL are inflammatory cytokines associated with alveolar bone loss in severe periodontitis and can be secreted by T cells (71–74). RT-qPCR results indicate that both wildtype and CD5KO CD4^+^ T cells upregulate Csf1 and Rankl mRNA transcription in response to stimulation across all stimulus types when compared to their respective unstimulated samples (**Supplementary Figure 7**). Additionally, CD4^+^ T cells from CD5KO mouse spleen have increased levels of CSF1 mRNA transcripts compared to WT CD4^+^ T cells when stimulated with CD3/28 antibody and supernatant from gingival epithelial cells treated with EC-LPS or mucosal epithelial cells treated with PG-LPS (**Figure 7**). In contrast, RANKL mRNA transcription was lower in CD5 knockout CD4^+^ T cells compared to wildtype when stimulated with CD3/28 antibodies and supernatant from gingival epithelial cells treated with PG-LPS or mucosal epithelial cells treated with PG-LPS (**Figure 7**).

## 4 Discussion

T cells play important roles in maintaining homeostasis in healthy gingiva and driving inflammatory responses in PD affected sites, and our current study provides further insights into how CD5, a regulator of T cell activity found on the surface of T cells, influences PD-induced T cell inflammation using an *in vitro* model to investigate the effects of *P. gingivalis* LPS on oral epithelial and immune cells. We chose to focus on the effect of *P. gingivalis* LPS on epithelial and immune cell function in the absence of other *P. gingivalis* antigens, such as gingipains, to better isolate the effects of PG-LPS and serve as a proof of concept of stimulating mouse oral epithelial cells to simulate oral inflammation before moving on to protein antigens or whole, attenuated *P. gingivalis*. PG-LPS has been used in other model systems and is an important trigger for oral diseases and inflammation initiation (75–77). Gingipains are important virulence factors of *P. gingivalis*, and their primary role is of proteolytic degradation of host tissues and immune components rather than serving as direct inflammatory stimuli (78, 79). Because our study focused specifically on characterizing the oral inflammatory response, we selected *P. gingivalis* LPS as our stimulus, as it has been useful to model inflammatory signaling in oral−immune systems (75–77).

In the present study, we first assessed how removal of CD5 affected T cell activation in our model (**Figure 1**). CD5 has been shown to attenuate TCR signaling through the recruitment of SHP-1 and E3-ubiquitin ligases CBL and CBLB to reduce the phosphorylation of proximal TCR signaling molecule Zap70 (80, 81). As observed in previous studies, removal of CD5 increased activation of CD4^+^ and CD8^+^ T cells when activated with CD3ϵ/28 antibodies for 48 hours and earlier time points (**Figure 2**; **Supplementary Figures 1**-**3**). Knockout T cells consistently had a statistically significant increase in CD69^+^ and CD25^+^ cell counts across several stimulation types. Thus, CD5 functions as an inhibitor of activation in this *in vitro* model. Previous studies have shown that LPS can affect T cell activation and differentiation (82). With this in mind, we questioned whether differences in activation kinetics between wildtype and knockout T cells were due to residual LPS found in the epithelial supernatant and cultured with the T cells. To address this, we measured residual LPS levels in the epithelial supernatants and did see residual levels (**Supplementary Figure 4**), but they do not appear to dramatically alter our results as indicated by the lack of changes in observed trends when T cells are treated with polymyxin B which blocks LPS across stimulation types and time points (**Figure 2**; **Supplementary Figures 1**–**3**).

We then measured how CD5 deletion affects T cell effector/memory and Treg differentiation in our *in vitro* model since CD5 influences T cell differentiation and these subsets have implications in PD pathogenesis. Before stimulating the splenic T cells, we observed increased counts of CD4^+^ effector T cells and CD8^+^ memory T cells among knockout splenocytes using flow cytometry (**Figure 3**). This was an observation that was to be expected as previous studies have shown that CD5 regulates T cell activity of effector cells and the survival of memory cells in the periphery. The initial increase in CD8^+^ memory counts persisted after 24 hours of activation was possibly due to the initial increased numbers. By day 2 of stimulation, all differences between wildtype and CD5 knockout have disappeared, but two-way ANOVA analysis indicated that variance due to genotype was statistically significant among CD8^+^ memory cell counts (**Figure 4A**). After 72 hours of stimulation, there were no differences in effector or memory counts in any of the PD stimulation types except for CD3ϵ/28 antibody only stimulation (increase among wildtype cells) (**Figure 4B**). However, two-way ANOVA analysis indicated that variance due to genotype was statistically significant among CD8^+^ effector and CD4 memory cell counts. Our observations suggest that removal of CD5 affects the kinetics of CD8^+^ memory T cell differentiation cells in this *in vitro* model as differences were observed at 24 hours but not at later time points (48 and 72 hours) (**Figures 3**; **4**). The initial delay in WT CD8+ memory counts compared to CD5KO may be explained by the regulatory role of CD5 in inhibiting TCR signaling during early activation. However, WT CD8^+^ effector counts catch up with CD5KO possibly due to sufficient stimulus signals from CD3/28 antibody stimulation and the epithelial supernatant over a sufficient amount of time. Additionally, we suggest that removal of CD5 possibly affects the kinetics of CD4^+^ and CD8^+^ effector differentiation as indicated by 2-way ANOVA analysis. Though, this effect is weak as indicated by the lack of statistically significant differences across almost all stimulation types at 24, 48, and 72 hours, while by 2-way ANOVA determined variance due to genotype was statistically significant in several instances (**Figures 3**; **4**). Previous studies have also shown that CD5 promotes Treg differentiation in the periphery, and they play important roles in regulating the immune response in several diseases including PD (83, 84). We did not see any differences in splenic Treg counts between wildtype and CD5 knockout before or after stimulation (1–3 days of stimulation) (**Supplementary Figure 1**). Treg promoting conditions, such as supplementation of IL-2 and TGFβ, were not incorporated into the current model. Perhaps, to more closely assess Treg function and differentiation in future directions IL-2 and TGFβ supplementation would allow researchers to investigate how CD5 signaling regulates these processes in PD.

On days 3 and 5, Th17 cell counts and cytokine production were measured to assess the inflammatory response by CD4^+^ T cells in this *in vitro* model. After 72-hours post stimulation, we observed an increase in Th17 counts among wildtype cells when stimulated only with antibodies (**Figure 5**). Though we saw an increase in Th17 cells in this stimulation type, we did not see any differences in IL17A levels in any of the stimulation types by 72-hours post stimulation (**Figure 6**). This discrepancy may be explained by decreased IL17A production by Th17 cells in general because we did not promote Th17 differentiation by adding IL-6 and TGFβ to the cell cultures and the levels of these cytokines produced surrounding T cells were not enough to complete terminal differentiation into the Th17 subset. TNF was the only cytokine that was significantly different at 3 days post stimulation in 3 of the stimulation types (CD3/28, GEC, and MEC) where wildtype had increased levels compared to knockout. This observation contradicts the expectation of hyperresponsiveness from CD5 knockout (or CD5 blockade treated) T cells seen in previous studies (62, 81, 85). TNF is a cytokine mainly produced by Th1 and CD8^+^ T cells, and it has been found to drive inflammation and osteoclast differentiation in PD (33). TNF is also a marker of a type 1 response that is aimed at eliminating bacterial pathogens. Comparative qPCR also detected differences in mRNA transcription of two other cytokines not included in the cytometric bead array, CSF1 and RANKL, between wildtype and CD5 knockout CD4^+^ T cells 3 days after stimulation (**Figure 7**). Both cytokines have been implicated in driving osteoclast differentiation and activity during PD, and T cells have been shown to secrete these cytokines during PD pathogenesis (46, 47, 71–73). Our results indicate that removal of CD5 increases CSF1 mRNA expression in CD4^+^ T cells while when stimulated with CD3/28 antibodies and supernatant from gingival epithelial cells treated with EC-LPS or mucosal epithelial cells treated with PG-LPS. Additionally, RANKL transcription in CD4^+^ T cells was decreased when CD5 was removed during stimulation with CD3/28 antibody and supernatant from gingival epithelial cells treated with PG-LPS or mucosal epithelial cells treated with PG-LPS. Taking everything together, removal of CD5 affects cytokine production but varies in direction depending on the cytokine with one proinflammatory cytokines being increased among knockouts (CSF1) and two being decreased (TNF and RANKL). Thus, we can conclude that CD5 modulates cytokine production but doesn’t cause global hyperactivation.

These results provide needed insights into how CD5 signaling affects T cell function specifically in the context of PD. However, we understand that there are some limitations in our study, including cell composition of the cultures and cell culturing conditions. In this study, we used mouse primary oral mucosal and gingival epithelial cells isolated from C57BL/6 mice which are most compatible with immune cells harvested from C57BL/6 mice. However, these cells lack the morphological and cell composition (tissue resident immune cells, osteoclasts/osteoblasts, blood vasculature, presence of teeth etc.) that are contained in the oral cavity that contribute to the complexity of PD pathogenesis. Additionally, the use of mouse splenocytes allows us to harvest a wide range of immune cell types (T cells, dendritic cells, B cells, NK cells, etc.) which are known to interact with each other regularly in the oral cavity, but their splenic origin may have a confounding effect on their functions observed in the assays performed. Lastly, stimulation of splenic T cells in this study ranges from 24 hours to 120 hours. This is enough time to understand how CD5 signaling affects early and late T cell activation. However, to better understand its effects on chronic PD one would need to assess immune cell responses weeks post bacterial challenge. Thus, any therapeutic implications from this study should be considered speculative as the *in vitro* model lacks key components of the periodontal microenvironment and no *in vivo* validation was performed. Nonetheless, we believe that this model can be used by researchers to understand the effects of *P. gingivalis* LPS on oral epithelial and immune cells. Using this model in the present study, we were able to characterize the functional differences between wildtype and CD5 knockout T cells and is the first step to understanding how CD5 regulates oral inflammation.

## Data Availability

The original contributions presented in the study are included in the article/**Supplementary Material**. Further inquiries can be directed to the corresponding author.
