# Supplementary figures and images for: Removal of CD5 on T cells alters their differentiation and cytokine production in an in vitro model investigating effects of P. gingivalis LPS on oral epithelial and immune cells
Source: Front Immunol. 2026 Jun 10;17:1761775. doi: 10.3389/fimmu.2026.1761775 (PMC13290719; doi:10.3389/fimmu.2026.1761775)

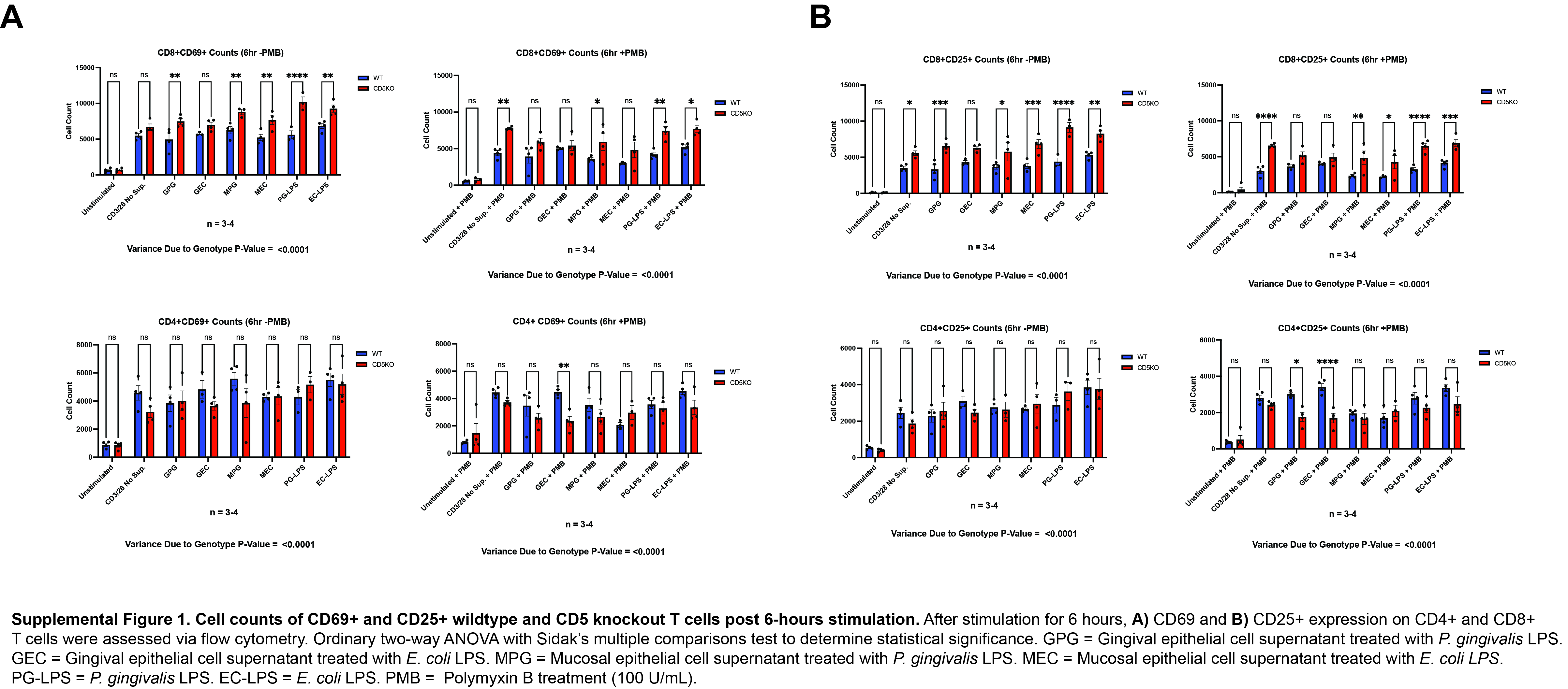

Supplement: Supplementary file 1 [file Image1.tif]

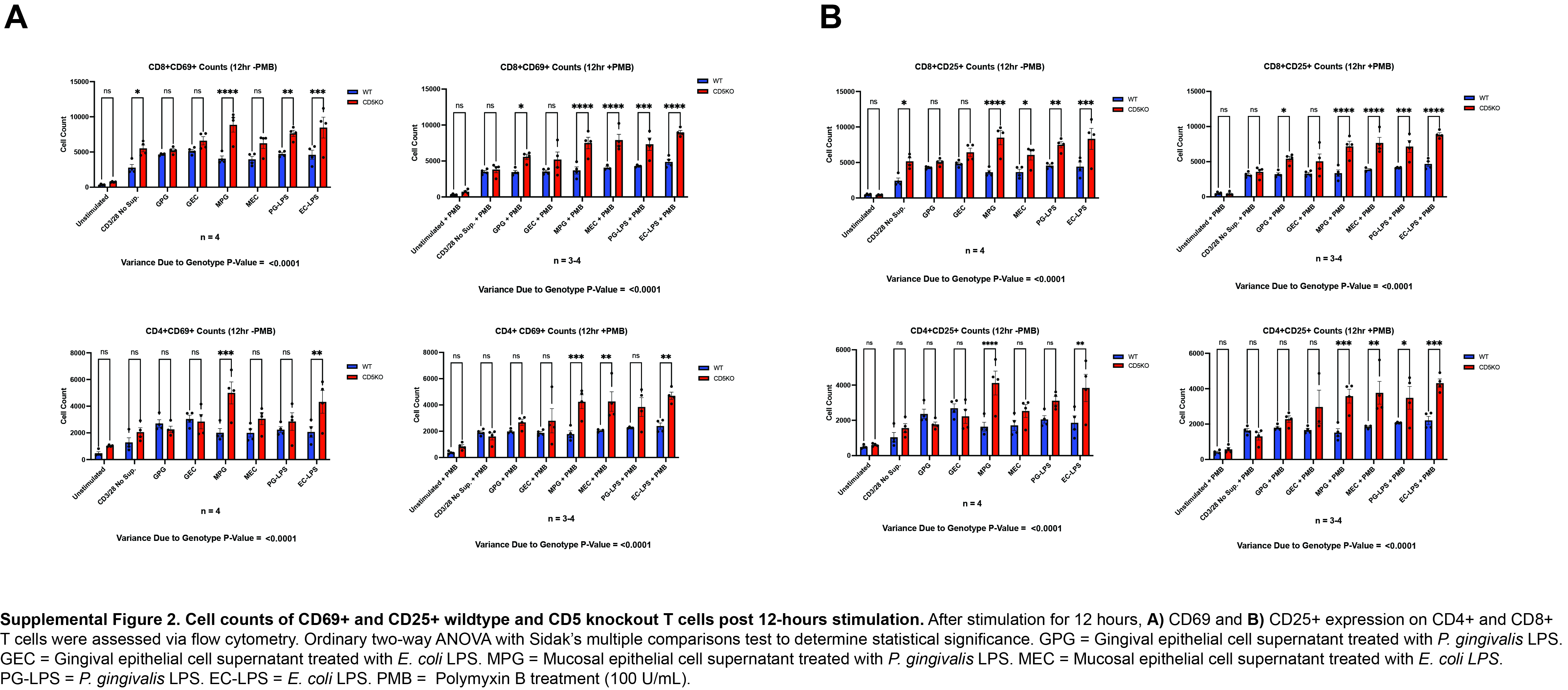

Supplement: Supplementary file 2 [file Image2.tif]

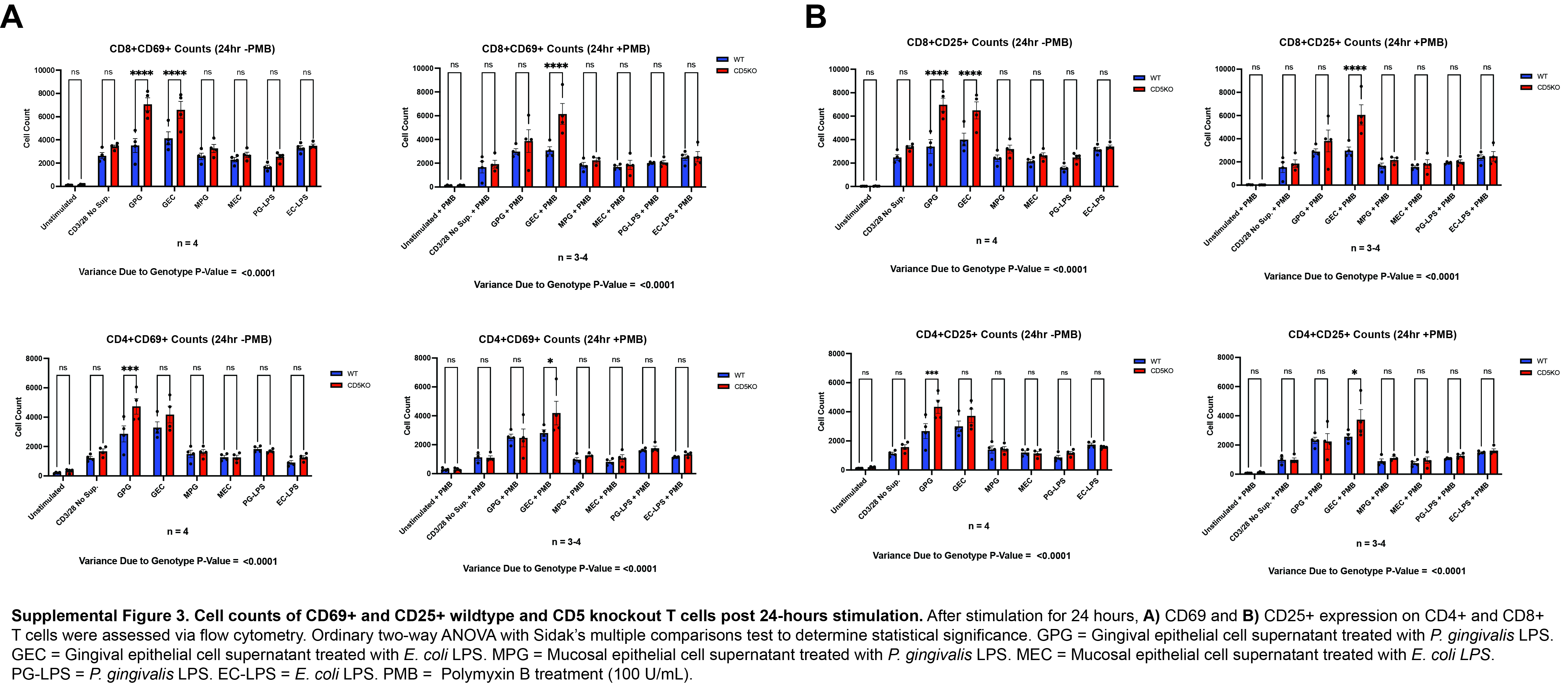

Supplement: Supplementary file 3 [file Image3.tif]

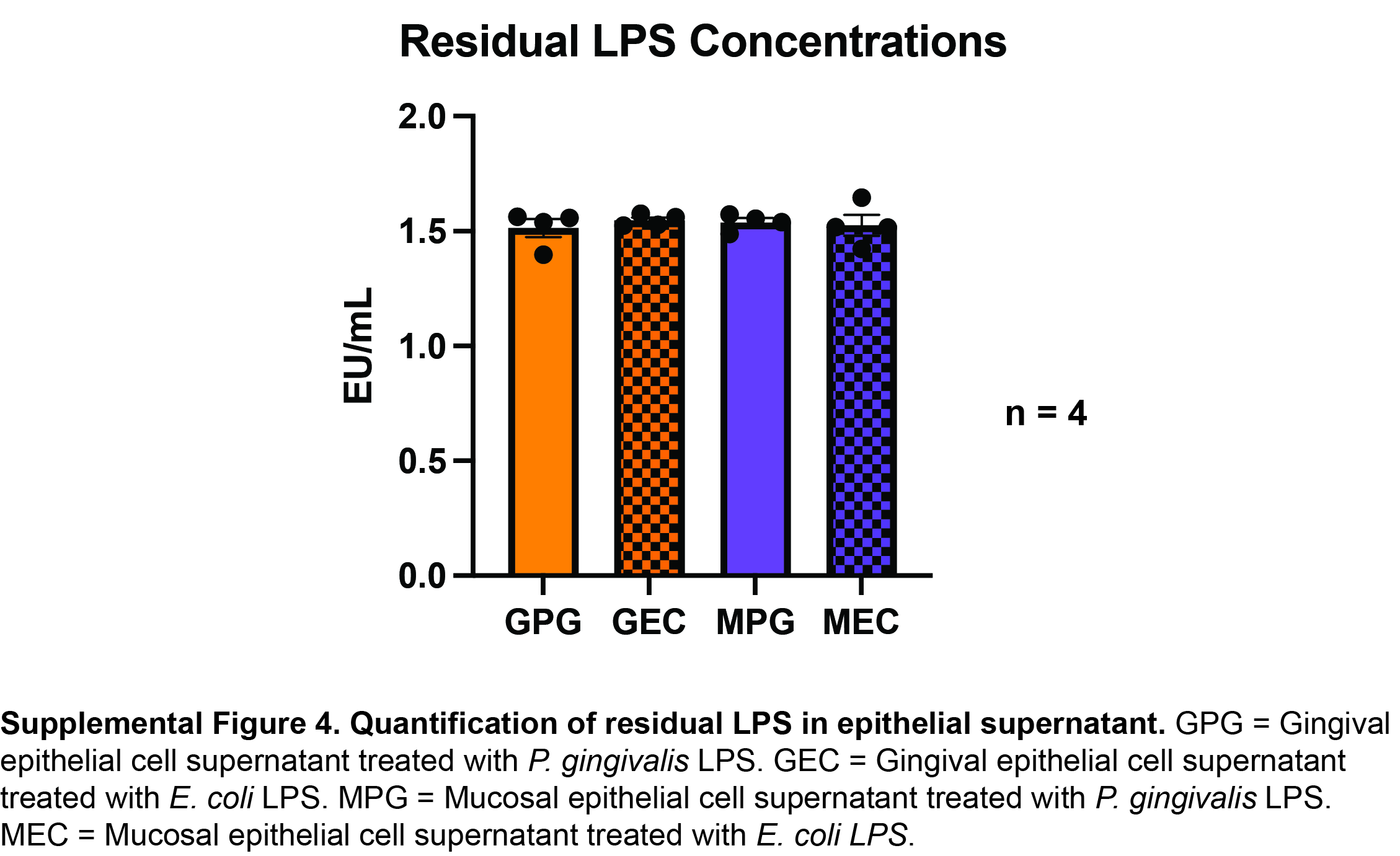

Supplement: Supplementary file 4 [file Image4.tif]

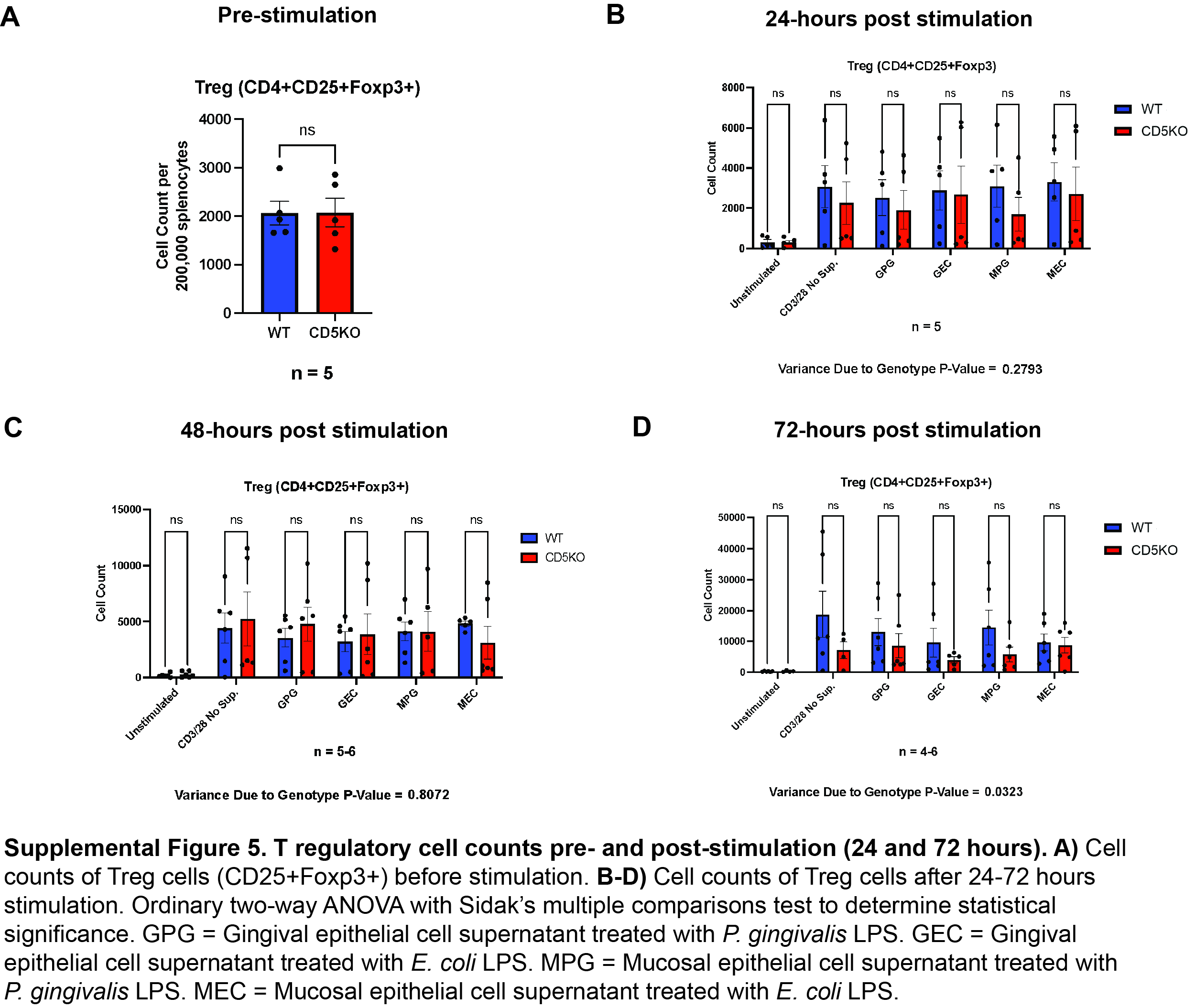

Supplement: Supplementary file 5 [file Image5.tif]

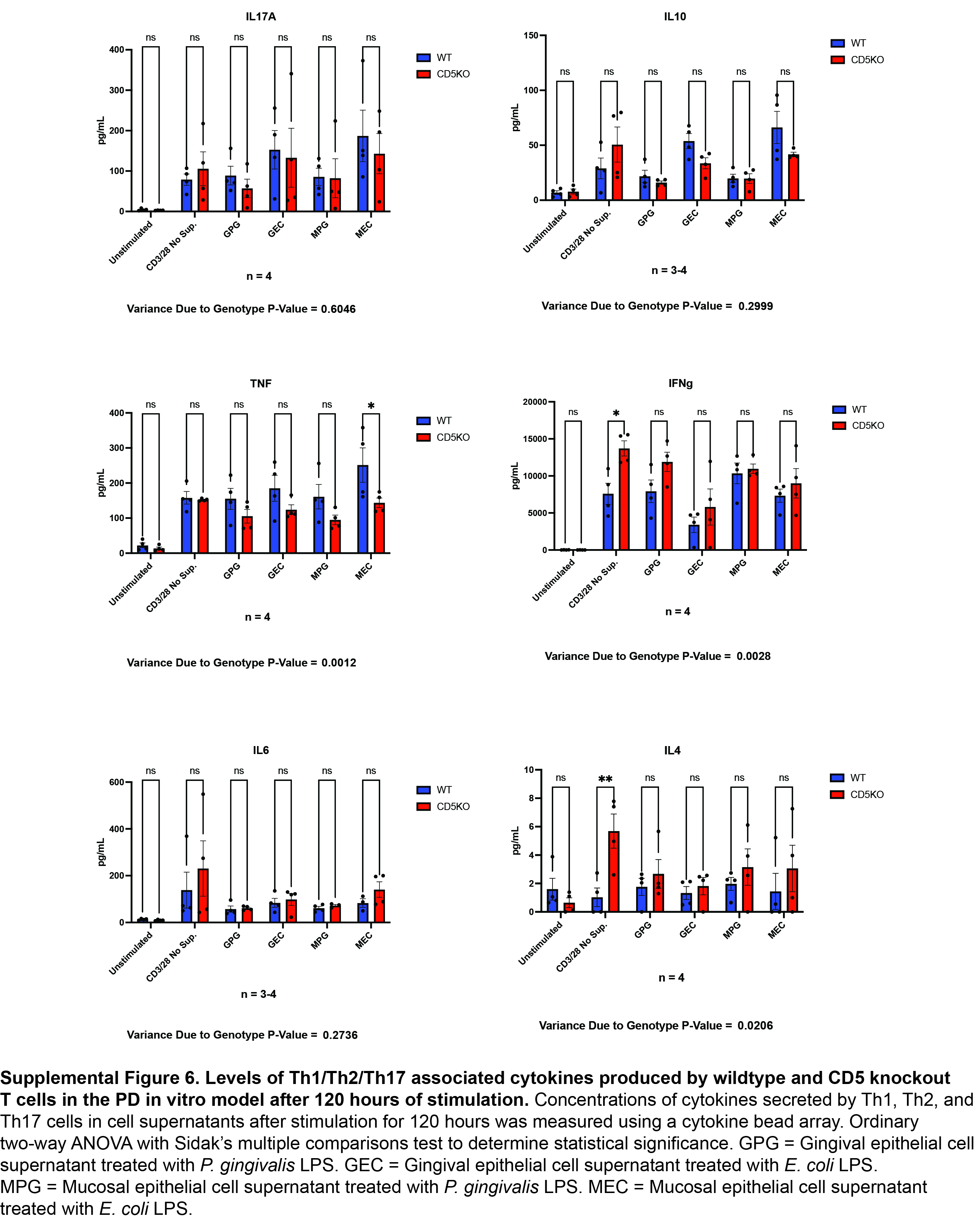

Supplement: Supplementary file 6 [file Image6.tif]

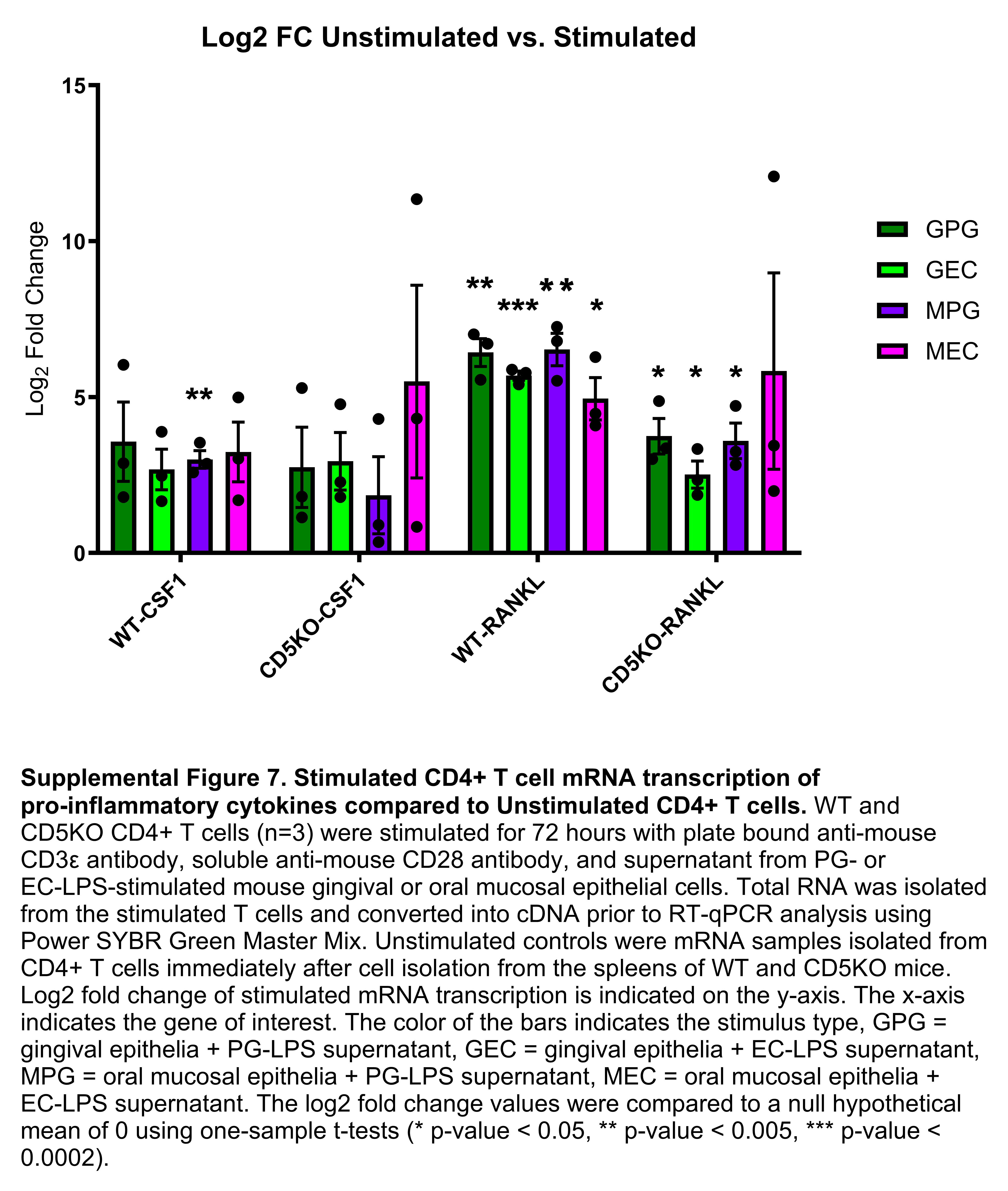

Supplement: Supplementary file 7 [file Image7.tif]
